# A Cross-Sectional Study on the Knowledge, Awareness, Myths, and Acceptability of Menstrual Cups Among Adolescent Girls in Nagamangala Taluk

**DOI:** 10.7759/cureus.110582

**Published:** 2026-06-10

**Authors:** Mahendra G, Jayanthi Sai Preethi, Manohar R

**Affiliations:** 1 Obstetrics and Gynecology, Adichunchanagiri Institute of Medical Sciences, Adichunchanagiri University, BG Nagara, IND; 2 Obstetrics and Gynecology, Mandya Institute of Medical Sciences, Mandya, IND

**Keywords:** acceptability, adolescents, knowledge, menstrual cups, menstrual hygiene

## Abstract

Introduction

Menstrual cups are a safe, cost-effective, and sustainable menstrual hygiene product; however, their awareness and acceptability among adolescents remain low due to socio-cultural barriers and misconceptions. The study aimed to assess the knowledge, awareness, myths, and acceptability of menstrual cups among adolescent girls and to evaluate factors influencing their willingness to use them.

Materials and methods

A community-based cross-sectional study was conducted among 812 adolescent girls aged 13-19 years in Nagamangala taluk, Karnataka, from February to April 2026. Data were collected using a pre-tested semi-structured questionnaire. Knowledge was categorized as adequate when >50% responses were correct. Associations were analyzed using the chi-squared test, with p < 0.05 considered statistically significant.

Results

Awareness of menstrual cups was observed in 308 (37.9%) participants. Adequate knowledge was present in 342 (42.1%), while myths and misconceptions were reported by 498 (61.3%). The most common misconception was fear of pain during insertion 362 (44.6%). Overall, 548 (67.5%) participants expressed willingness to use menstrual cups. Awareness increased significantly with age (χ² = 9.82, p = 0.002). Willingness was significantly higher among those aware, 220 (71.4%), compared to those not aware, 328 (65.1%) (χ² = 4.21, p = 0.040). Adequate knowledge was associated with higher acceptability (χ² = 14.7, p < 0.001), while the presence of myths was associated with lower willingness (χ² = 41.6, p < 0.001).

Conclusion

Despite low awareness and prevalent misconceptions, the acceptability of menstrual cups was relatively high, particularly after exposure to appropriate information. Strengthening menstrual health education and addressing myths may improve the adoption of menstrual cups among adolescents.

## Introduction

Menstruation is a normal physiological process experienced by adolescent girls and women of reproductive age; however, it continues to be surrounded by stigma, taboos, and misinformation, particularly in low- and middle-income countries such as India [1,2]. Inadequate menstrual hygiene management (MHM) has significant implications for physical health, psychological well-being, school attendance, and overall quality of life among adolescents [3]. Limited access to accurate information, coupled with socio-cultural restrictions, often leads to unsafe practices and perpetuates myths and misconceptions related to menstruation and its management [4,5].

Globally, menstrual health has been recognized as an essential component of adolescent reproductive health and gender equity and emphasize the need for comprehensive menstrual education and access to safe menstrual products [6,7]. In India, despite various governmental initiatives aimed at improving menstrual hygiene, disposable sanitary pads remain the predominant choice [8]. Awareness and uptake of alternative menstrual products, particularly menstrual cups, remain relatively low [9]. Cultural beliefs, apprehension regarding internal products, fear of pain, and concerns related to virginity act as significant barriers to their acceptance [10].

Menstrual cups are reusable, bell-shaped devices made of medical-grade silicone, rubber, or latex that collect menstrual blood [11]. They offer several advantages, including cost-effectiveness, long-term usability, and environmental sustainability [12]. Evidence suggests that menstrual cups are safe, associated with fewer adverse effects, and result in high user satisfaction following initial adaptation [13]. Despite these benefits, their utilization among adolescent girls remains limited, especially in rural and semi-urban settings, where awareness is low and misconceptions are widespread [14].

In such contexts, myths related to insertion, discomfort, infection, and perceived impact on virginity significantly influence attitudes toward menstrual cup use [15]. Additionally, lack of structured menstrual education, inadequate counselling, and limited exposure to reliable information further hinder informed decision-making among adolescents [16]. Nagamangala taluk, representative of many rural settings in India, faces similar challenges in adolescent health education and menstrual hygiene awareness. Therefore, this study aimed to assess the knowledge, awareness, myths, and acceptability of menstrual cups among adolescent girls in Nagamangala taluk and to evaluate factors influencing their willingness to use this sustainable menstrual hygiene product.

## Materials and methods

Study design and setting

This community-based cross-sectional study was conducted among adolescent girls in Nagamangala taluk, Mandya district, Karnataka, India, over a period of three months from February 2026 to April 2026.

Ethical considerations

Ethical approval was obtained from the Institutional Ethics Committee (Ref No: AIMS/IEC/037/2026). Permission was obtained from school authorities prior to data collection. Written informed consent was obtained from parents or guardians for participants below 18 years, along with assent from the adolescents. Confidentiality and anonymity of all participants were strictly maintained throughout the study.

Study participants

The study population comprised school-going adolescent girls aged 13-19 years who had attained menarche. Participants who were willing to participate and provided assent/consent were included in the study. Those who had not attained menarche, were seriously ill at the time of data collection, or did not provide consent/assent were excluded.

Sample size and sampling technique

The sample size was calculated using the formula for a single proportion:

\[n=\frac{Z^2\times p\times q}{d^2}\]

where Z=1.96 for 95% confidence interval, p = prevalence from previous study, q=1−p, and d = absolute precision. Based on the study by Abraham et al., the prevalence of menstrual cup usage was 1.07% [17]. Taking p=0.0107, q=0.9893, and assuming a precision of d=0.01, the calculated sample size was approximately 406. Considering design effect and feasibility, a total of 812 participants were included in the study. A simple random sampling technique was used to select participants from schools until the required sample size was achieved.

Data collection tool and procedure

Data were collected using a pre-designed, pre-tested, semi-structured questionnaire. The tool included four sections: socio-demographic details, knowledge and awareness regarding menstrual cups, myths and misconceptions, and acceptability, including willingness to use menstrual cups. The questionnaire was prepared in English, translated into Kannada, and back-translated to ensure accuracy. Data collection was carried out through face-to-face interviews in a private setting.

Operational definitions

Operational definitions were applied for key variables. Awareness was defined as having heard about menstrual cups. Knowledge was assessed using multiple items related to usage, duration, cleaning, and cost-effectiveness, and categorized as adequate when more than 50% of responses were correct and inadequate when 50% or fewer responses were correct. Myths and misconceptions were considered present if participants reported one or more incorrect beliefs. Acceptability was defined as the willingness to use menstrual cups.

Statistical analysis

Data were entered into Microsoft Excel (Microsoft, Redmond, Washington) and analyzed using Statistical Package for Social Sciences (SPSS) version 26.0 (IBM Inc., Armonk, New York). Descriptive statistics such as frequencies and percentages were used to summarize categorical variables. The association between awareness, knowledge, myths, and acceptability with relevant variables was assessed using the chi-squared test. A p-value of <0.05 was considered statistically significant.

## Results

The study included 812 adolescent girls, with the majority aged 16-17 years, 366 (45.1%), followed by 13-15 years, 312 (38.4%), while 18-19 years comprised 134 (16.5%). Most participants were from rural areas, 562 (69.2%), whereas 250 (30.8%) belonged to semi-urban areas (Table 1).

**Table 1:** Socio-demographic characteristics (n = 812) Socio-demographic characteristics of study participants (n = 812). Data are presented as N (%). No statistical test was applied.

Variable	Category	N (%)
Age	13-15 years	312 (38.4%)
	16-17 years	366 (45.1%)
	18-19 years	134 (16.5%)
Residence	Rural	562 (69.2%)
	Semi-urban	250 (30.8%)

Knowledge regarding menstrual cups was suboptimal, with 356 (43.8%) aware of their reusable nature. Knowledge of cost-effectiveness was observed in 310 (38.2%), and duration of use in 298 (36.7%), while correct cleaning practices were known to only 264 (32.5%), indicating gaps in practical understanding (Table 2).

**Table 2:** Knowledge parameters regarding menstrual cups (n = 812) Knowledge parameters regarding menstrual cups among participants (n = 812). Data are presented as N (%). No statistical test was applied.

Parameter	N (%)
Knows reusable nature	356 (43.8%)
Knows duration of use	298 (36.7%)
Knows cleaning method	264 (32.5%)
Knows cost-effectiveness	310 (38.2%)

Misconceptions were widely prevalent, with fear of pain during insertion reported by 362 (44.6%). Concerns regarding impact on virginity were noted in 304 (37.4%), while 266 (32.8%) believed the cup could get stuck. Fewer participants, 188 (23.1%), reported fear of infection (Table 3).

**Table 3:** Distribution of myths and misconceptions (n = 812) Distribution of myths and misconceptions regarding menstrual cups (n = 812). Data are presented as N (%). No statistical test was applied.

Myth / misconception	N (%)
Pain during insertion	362 (44.6%)
Affects virginity	304 (37.4%)
Cup may get stuck	266 (32.8%)
Causes infection	188 (23.1%)

Overall awareness was limited, with only 308 (37.9%) participants aware of menstrual cups, while 504 (62.1%) were not aware. Adequate knowledge was present in 342 (42.1%), and misconceptions were reported by 498 (61.3%). Despite this, 548 (67.5%) participants expressed willingness to use menstrual cups compared to 264 (32.5%) who were unwilling (Table 4).

**Table 4:** Distribution of knowledge, awareness, myths, and acceptability (n = 812) Distribution of awareness, knowledge level, myths/misconceptions, and acceptability of menstrual cups (n = 812). Data are presented as N (%). No statistical test was applied.

Variable	Category	N (%)
Awareness	Aware	308 (37.9%)
	Not aware	504 (62.1%)
Knowledge level	Adequate	342 (42.1%)
	Inadequate	470 (57.9%)
Myths/ misconceptions	Present	498 (61.3%)
	Absent	314 (38.7%)
Acceptability (willingness)	Willing	548 (67.5%)
	Not willing	264 (32.5%)

Awareness increased significantly with age, from 80 (25.6%) in the 13-15 years group to 160 (43.7%) in the 16-17 years group and 68 (50.7%) in the 18-19 years group, with a statistically significant association (χ² = 9.82, p = 0.002) (Table 5).

**Table 5:** Awareness of menstrual cups by age group (n = 812) Association between age group and awareness of menstrual cups (n = 812). Data are presented as N (%). Statistical test used: chi-squared test. A p-value < 0.05 was considered statistically significant.

Age group	Aware, N (%)	Not aware, N (%)	χ²	p-value
13-15 years (n = 312)	80 (25.6%)	232 (74.4%)	9.82	0.002
16-17 years (n = 366)	160 (43.7%)	206 (56.3%)		
18-19 years (n = 134)	68 (50.7%)	66 (49.3%)		

Willingness to use menstrual cups was higher among those who were aware, 220 (71.4%), compared to those not aware, 328 (65.1%) (χ² = 4.21, p = 0.040). Similarly, participants with adequate knowledge showed greater willingness, 256 (74.9%) versus 292 (62.1%) with inadequate knowledge (χ² = 14.7, p < 0.001). In contrast, willingness was lower among those with misconceptions, 276 (55.4%), compared to those without misconceptions, 272 (86.6%) (χ² = 41.6, p < 0.001) (Table 6).

**Table 6:** Factors associated with acceptability of menstrual cups (n = 812) Factors associated with acceptability (willingness to use) menstrual cups among participants (n = 812). Data are presented as N (%). Statistical test used: chi-squared test. A p-value < 0.05 was considered statistically significant.

Variable	Category	Willing, N (%)	Not willing, N (%)	χ²	p-value
Awareness	Aware (n = 308)	220 (71.4%)	88 (28.6%)	4.21	0.040
	Not aware (n = 504)	328 (65.1%)	176 (34.9%)		
Knowledge	Adequate (n = 342)	256 (74.9%)	86 (25.1%)	14.7	<0.001
	Inadequate (n = 470)	292 (62.1%)	178 (37.9%)		
Myths	Present (n = 498)	276 (55.4%)	222 (44.6%)	41.6	<0.001
	Absent (n = 314)	272 (86.6%)	42 (13.4%)		

## Discussion

The present study demonstrates that awareness of menstrual cups among adolescent girls remains limited, with only 308 (37.9%) participants having heard about them. This finding is consistent with earlier Indian studies, where menstrual cups were among the least recognized menstrual hygiene products. Kakani et al. also reported low awareness despite adequate general menstrual knowledge, while Kaur et al. observed that sanitary pads dominated awareness compared to alternative products [18,19]. These similarities suggest that menstrual cup information has not been adequately integrated into adolescent health education programs across different regions of India.

A significant age-wise gradient in awareness was observed in the present study, increasing from 80 (25.6%) in early adolescents to 68 (50.7%) in late adolescents (χ² = 9.82, p = 0.002). This trend aligns with findings by Patil VV et al., who reported improved awareness of alternative menstrual products with increasing age [20]. The likely explanation includes greater exposure to formal education, peer discussions, and digital media among older adolescents. This highlights the importance of introducing menstrual health education at earlier ages to bridge this awareness gap.

Despite low baseline awareness, the acceptability of menstrual cups was relatively high. In the present study, 220 (71.4%) of aware participants and 328 (65.1%) of previously unaware participants expressed willingness to use menstrual cups after receiving information. These findings are comparable to those reported by Beksinska et al., where educational interventions significantly improved acceptability and sustained usage [21]. This indicates that lack of awareness, rather than resistance, is the primary barrier, and that appropriate education can substantially improve adoption.

Misconceptions were highly prevalent, with 362 (44.6%) reporting fear of pain during insertion and 304 (37.4%) expressing concerns regarding virginity. Similar socio-cultural barriers have been documented in studies from Delhi and rural Maharashtra, where internal menstrual products were often linked to stigma and cultural taboos [22]. Importantly, the present study demonstrated a strong association between myths and reduced acceptability, with willingness significantly lower among those with misconceptions 276 (55.4%) compared to those without 272 (86.6%) (χ² = 41.6, p < 0.001). Comparable findings were reported by Sharma et al., emphasizing that myths and negative perceptions are key determinants of poor uptake [22]. These findings underscore the need for targeted, culturally sensitive educational interventions focusing on myth reduction and accurate information dissemination to improve menstrual cup acceptance among adolescents.

Strengths and limitations

The present study has several strengths. First, it included a relatively large sample of 812 adolescent girls drawn from rural and semi-urban settings, providing robust estimates of awareness, knowledge, misconceptions, and acceptability of menstrual cups. Second, the study assessed multiple dimensions of menstrual cup perception simultaneously, allowing a comprehensive evaluation of factors influencing acceptability. Third, the use of a pre-tested structured questionnaire and standardized face-to-face data collection enhanced data quality and consistency. Furthermore, the inclusion of participants across different adolescent age groups enabled assessment of age-related differences in awareness and acceptability.

However, certain limitations should be acknowledged. The cross-sectional design precludes establishing causal relationships between awareness, knowledge, misconceptions, and willingness to use menstrual cups. The findings were based on self-reported responses and may therefore be subject to recall bias and social desirability bias. The study was limited to school-going adolescents and did not include out-of-school girls, which may restrict the generalizability of the findings. In addition, although willingness to use menstrual cups was assessed, actual adoption, continued usage, and long-term experiences were not evaluated.

## Conclusions

The present study highlights that awareness and knowledge regarding menstrual cups among adolescent girls were limited, with a high prevalence of myths and misconceptions influencing perceptions. Despite this, a substantial proportion of participants demonstrated willingness to use menstrual cups, particularly following exposure to appropriate information. Acceptability was significantly associated with better knowledge and absence of misconceptions, indicating that misinformation remains a key barrier to adoption. These findings emphasize the need for structured, age-appropriate, and culturally sensitive menstrual health education focusing on awareness generation and myth reduction. Integrating such interventions into school-based and community health programs may improve acceptance and promote the use of menstrual cups as a safe, cost-effective, and sustainable menstrual hygiene option.

## References

[1] Selva C, Butragueño MN, de Miquel C (2026). Gender equity and menstrual justice: a psychosocial review on stigma and discrimination in access to reproductive health. Reprod Health.

[2] Vinod A, Thakur JS, Rajagopal V (2025). A study of menstrual health perceptions, practices and prevalence of premenstrual symptoms in schoolgoing adolescent girls in Chandigarh. Int J Community Med Public Health.

[3] Betsu BD, Medhanyie AA, Gebrehiwet TG, Wall LL (2024). Menstrual hygiene management interventions and their effects on schoolgirls' menstrual hygiene experiences in low and middle countries: a systematic review. PLoS One.

[4] Tshivule MZ, Rasweswe MM, Mothiba TM, Bopape MA (2025). Factors influencing menstrual hygiene knowledge, attitudes, and practices among adolescent girls in African rural schools: scoping review. Front Reprod Health.

[5] Castro S, Czura K (2025). Cultural taboos and misinformation about menstrual health management in rural Bangladesh. World Dev.

[6] Sommer M, Hennegan J, Muralidharan A, Kabiru CW, Mahon T, Phillips-Howard PA (2025). Adolescent menstrual health must go beyond pads. BMJ.

[7] Joshi K, Mendhe D (2025). Navigating menstrual health and hygiene: challenges and solutions for adolescent girls. J Pharm Bioallied Sci.

[8] Kumari S, Muneshwar KN (2023). A review on initiatives for promoting better menstrual hygiene practices and management in India. Cureus.

[9] Davile M, Gangane N, Khan MF, Dange S, Mundle S (2024). Exploring menstrual hygiene practices and awareness of menstrual cups among nursing professionals: a cross-sectional survey. Cureus.

[10] Botello-Hermosa A, González-Cano-Caballero M, Guerra-Martín MD, Navarro-Pérez CF, Arnedillo-Sánchez S (2024). Perceptions, beliefs, and experiences about the menstrual cycle and menstruation among young women: a qualitative approach. Healthcare (Basel).

[11] van Eijk AM, Zulaika G, Lenchner M (2019). Menstrual cup use, leakage, acceptability, safety, and availability: a systematic review and meta-analysis. Lancet Public Health.

[12] Behera SM, Behera P, Pal D, Malla PS, Patro BK, Rao EV (2025). Menstrual cup use among nulliparous women: breaking taboos and empowering communities - a scoping review. Indian J Community Med.

[13] Singh R, Agarwal M, Sinha S, Chaudhary N, Sinha HH, Anant M (2022). Study of adaptability and efficacy of menstrual cups in managing menstrual health and hygiene: a descriptive longitudinal study. Cureus.

[14] Bhattarai A, Shrestha VL, Bist A (2025). Knowledge, attitude and use of menstrual cup among females of Siddharthanagar Municipality, Nepal: a community-based cross-sectional study. BMJ Open.

[15] Sarathivarman S, Krishnan P, Ravi K, Leelabai BS, Kanagaraj TS, Ayyavoo S, Periasamy P (2025). Knowledge, perceptions, and acceptance of menstrual cups among medical and engineering students in Chennai: a comparative cross-sectional study. J Pharm Bioallied Sci.

[16] Holmes K, Curry C, Sherry Sherry (2021). Adolescent menstrual health literacy in low, middle and high-income countries: a narrative review. Int J Environ Res Public Health.

[17] Abraham R, Rajan MP, Sajithamony Sajithamony (2023). Knowledge, acceptability and misconceptions regarding menstrual cup among college students of Kerala: a cross-sectional study. Indian J Public Health Res Dev.

[18] Kakani CR, Bhatt JK (2017). Study of adaptability and efficacy of menstrual cup in managing menstrual health and hygiene. Int J Reprod Contracept Obstet Gynecol.

[19] Kaur R, Kaur K, Kaur R (2018). Menstrual hygiene, management, and waste disposal: practices and challenges faced by girls/women of developing countries. J Environ Public Health.

[20] Patil VV, Udgiri R (2016). Menstrual hygienic practices among adolescent girls of rural North Karnataka region, India. Int J Community Med Public Health.

[21] Beksinska ME, Smit J, Greener R, Todd CS, Lee ML, Maphumulo V, Hoffmann V (2015). Acceptability and performance of the menstrual cup in South Africa: a randomized crossover trial comparing the menstrual cup to tampons or sanitary pads. J Womens Health (Larchmt).

[22] Sharma S, Mehra D, Brusselaers N, Mehra S (2020). Menstrual hygiene preparedness among schools in India: a systematic review and meta-analysis of system-and policy-level actions. Int J Environ Res Public Health.

